# The Effectiveness of Curcumin in Treating Oral Mucositis Related to Radiation and Chemotherapy: A Systematic Review

**DOI:** 10.3390/antiox13101160

**Published:** 2024-09-25

**Authors:** Gianna Dipalma, Angelo Michele Inchingolo, Giulia Latini, Laura Ferrante, Paola Nardelli, Giuseppina Malcangi, Irma Trilli, Francesco Inchingolo, Andrea Palermo, Alessio Danilo Inchingolo

**Affiliations:** 1 Department of Interdisciplinary Medicine, University of Bari “Aldo Moro”, 70124 Bari, Italy; giannadipalma@tiscali.it (G.D.); angeloinchingolo@gmail.com (A.M.I.); dr.giulialatini@gmail.com (G.L.); lauraferrante79@virgilio.it (L.F.); drnardellipaola@gmail.com (P.N.); trilliirma@gmail.com (I.T.); ad.inchingolo@libero.it (A.D.I.); 2 College of Medicine and Dentistry, Birmingham B4 6BN, UK; andrea.palermo2004@libero.it

**Keywords:** curcumin, oral mucositis, oral disease, radiotherapy, chemotherapy, oral health, ROS

## Abstract

Chemotherapy (CT) and radiation therapy (RT), while effective against cancer, often cause severe side effects, such as oral mucositis and other oral diseases. Oral mucositis, characterized by inflammation and ulceration of the oral mucosa, is one of the most painful side effects that can reduce quality of life and limit cancer treatment. Curcumin, a polyphenol from *Curcuma longa*, has garnered attention for its anti-inflammatory, antioxidant, and anti-carcinogenic properties, which protect the oral mucosa by reducing oxidative stress and modulating inflammation. This study reviews the therapeutic potential of curcumin in preventing and managing oral mucositis caused by CT and RT. Clinical trials show curcumin’s effectiveness in reducing the incidence and severity of oral mucositis. Although curcumin supplementation appears to be a promising and cost-effective approach for mitigating oral complications in cancer patients, further clinical trials are needed to confirm its efficacy and optimize dosing strategies.

## 1. Introduction

Today, according to estimates reported by the International Agency for Research on Cancer as part of the GLOBOCAN project, one in five people will be affected by cancer during their lifetime, with a higher mortality prevalence in the male sex compared to the female sex (4:3) [1]. Worldwide, lung cancer is the cancer with the highest incidence, exceeding in percentage those of breast, colorectal, prostate, stomach, head, and neck cancers [1].

Because of the world’s growing and aging population, it is estimated that there will be 35 million new cases of cancer in 2050, up from 20 million in 2022 [1,2].

**Figure 1:**
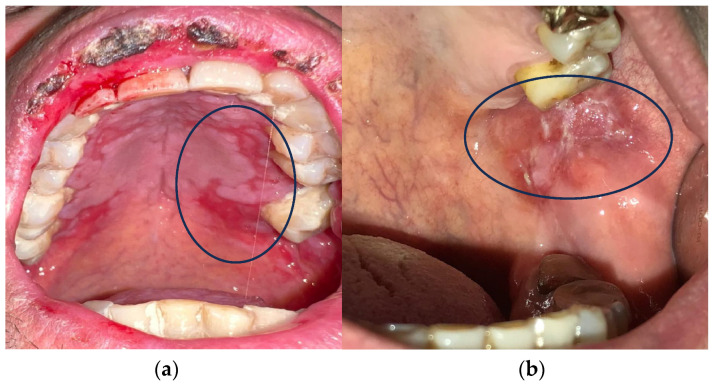
(**a**,**b**) Oral mucositis induced by radiotherapy and chemotherapy.

Cancer treatment is a major challenge in modern medicine. Based on different parameters, there are many therapeutic treatment options [3,4,5,6]. Systemic CT and RT are the most popular because they are most effective in highly invasive cancers [7]. The occurrence of oral mucositis (OM) and gastrointestinal mucositis is one of the side effects of CT and RT (Figure 1) [8,9,10].

**Figure 2:**
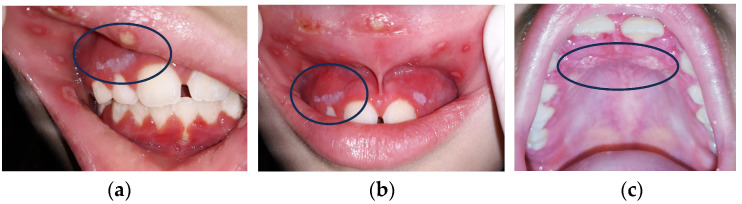
(**a**–**c**) Three kinds of oral lesions in immunodepressed girl with neck cancer.

OM incidence rates in CT patients are variable, depending on chemotherapy protocols (20–40% in conventional CHT, 80% in high-dose CT, 75–100% in HSCT, and consistently after RT in head and neck squamous cell cancer patients) (Figure 2a–c) [11,12,13,14,15].

**Figure 3:**
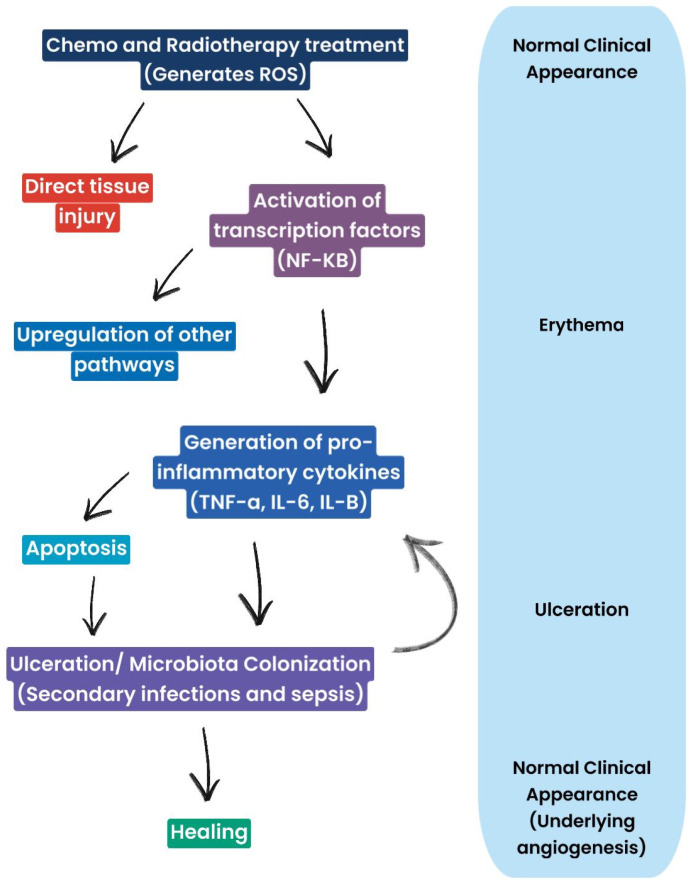
Diagram representing mucosal cells and clinical manifestations of oral mucositis: ROS generation leads, on the one hand, to tissue damage and inflammation and, on the other hand, triggers pathways involved in repair and healing. Therefore, the healing process is not strictly linear, but it involves a balance between damage and healing processes.

Oral CT mucositis (OCT) occurs after 4–5 days and disappears approximately one month after cessation of administration, depending on the severity of the mucosal lesions and the patient’s overall health [16,17] (Figure 2). OM from RT (RTMO) also occurs a few days later and becomes chronic, as it can persist for 3–4 weeks after treatment [18,19,20,21]. Healing occurs 6–8 weeks after the end of therapy [8,22,23,24]. OM is highly disabling and has a significant impact on the patient’s quality of life. It manifests itself with painful lesions that make feeding and oral hygiene difficult and promotes the onset of local and systemic bacterial infections in immunodepressed and immunocompromised cancer patients [25]. It leads to the reduction or even discontinuation of CT and RT, reducing the effectiveness of antineoplastic therapy (Figure 3).

Clinical manifestations of CT- and RT-induced inflammation follow a typical sequence. Initially, erythema is observed, indicating inflammation and increased vascular permeability. This may progress to ulceration, which represents more advanced damage, with impairment of oral mucosal integrity. In the final stage, healing is observed, with restoration of mucosal integrity and angiogenesis. Ulcerations are often complicated by microbial colonization, which can lead to secondary infections and worsen the overall clinical picture.

Nutritional and psychological status are impaired, as there is also an alteration of taste [26]. To date, there are no real guidelines for the OM treatment [27,28]. Synthetic mouthwashes (chlorhexidine and mucosamine) are commonly prescribed [29,30]. Chlorhexidine gluconate, the most prescribed mouthwash for the OM treatment, although having good potential for resolution of CT and RT lesions, has manifested side effects, such as dehydration and burning, which increase the likelihood of bacterial infection of the affected mucosa itself. In addition, the presence of alcohol in mouthwashes increases and/or stimulates pain [7,31,32,33]. A new therapeutic approach is the use of mouthwashes based on hyaluronic acid supplemented with amino acids such as mucosamine. They have shown antioxidant properties, acting directly on reactive oxygen species (ROS), scavenging and blocking them. They have shown good ability to reduce pain and inflammation and to regenerate damaged oral mucosa [18,34]. In more advanced stages, cryotherapy and photobiomodulation (PBM) protocols, as well as topical analgesics based on morphine, benzocaine, menthol, and cortisone, are also used to reduce pain symptoms [35,36,37].

### 1.1. Curcumin: Mechanisms of Action

The use of herbal medicines is a focus in the OM treatment [22,38,39]. In particular, recent studies have provided information on the complex biological mechanisms of phytotherapies, confirming their prophylactic and therapeutic properties in various diseases of the oral cavity (mucositis, gingivitis, aphthous stomatitis, caries, periodontitis, oral cancer, oral candidiasis, oral submucosal fibrosis, precancerous lesions, etc.) [40,41,42,43]. One of the most researched substances is curcumin, as a phytotherapeutic agent, and has gained special prominence. Curcumin (diferuloylmethane) is derived from the root of Curcuma longa, an Asian plant of the Zingiberaceae family, and is considered the most active component of turmeric [44,45]. Since ancient times, curcumin has been recognized for its therapeutic properties (healing, antioxidant, antifungal, anti-inflammatory, antidepressant, adjuvant to chemo and radiation therapies, hypoglycemic), supported today by multiple scientific studies [46,47,48,49,50,51]. Its antioxidant role is particularly important in the context of oral mucositis, where curcumin helps mitigate oxidative stress, one of the key factors in mucosal damage caused by chemotherapy and radiation therapy. Curcumin’s ability to neutralize free radicals and reduce oxidative damage plays a critical role in preserving the integrity of the oral mucosa, thereby reducing the severity and duration of mucositis.

Using curcumin showed an interesting phytotherapeutic result in CTOM and RTOM, with excellent tolerance also shown in pediatric cancer patients [40,52,53].

**Figure 4:**
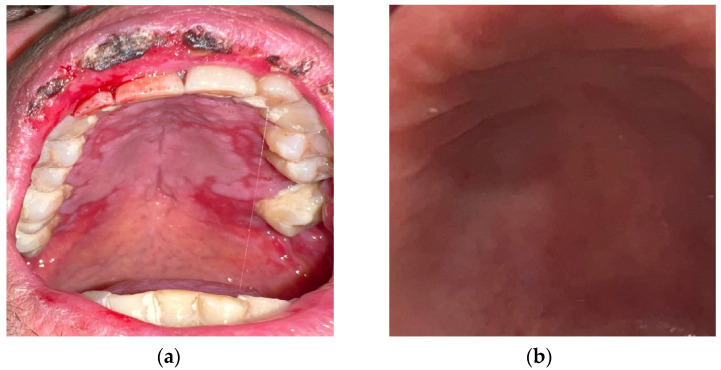
(**a**) Post-chemotherapy palatal mucositis; (**b**) detail of the palatal mucosa after treatment with curcumin gel.

A variety of formulations have been used (tablets, films, mouthwashes, gels, sprays, pastes, and chewing gum) [54]. Topical therapy, especially gel formulation, is better tolerated and has fewer side effects compared to systemic administration, is easier to apply, increases contact time and thus absorption, enhancing the soothing and re-epithelializing effects [55,56,57] (Figure 4a,b). In all cases, patients with CTOM and RTOM who underwent curcumin therapy manifested lower exposure to mucositis onset, reduced pain symptoms, and faster recovery, without side effects [54,58,59,60,61]. In patients treated with curcumin, the mean visual analog scale (VAS) scores were significantly lower after 3–4 weeks of radiotherapy and at post-radiotherapy visits compared to patients treated with other therapies [22,62,63].

The reduction in pain symptoms is due not only to better and faster tissue healing, but also, at high doses, to the release of serotonin, dopamine, and norepinephrine, with reduced pain transmission pathways and increased morphine efficacy [64,65,66]. Its pleiotropic nature ensures phytotherapeutic properties. In fact, by interfering with several biological processes and the release of inflammatory factors, including ROS, C-reactive proteins, interleukins (ILs), cytokines, transforming growth factor-β (TGF-β), cyclooxygenase-2 (COX-2), nuclear factor kappa B (NF-κB), tumor necrosis factor-α (TNF-α), it modulates the oxidation-reduction phenomena [67,68,69,70]. Thus, by acting on the various factors involved in the complex mechanism of chain reactions now recognized in the onset of OM, it ensures their control and therapeutic action [22,52,71,72].

Moreover, curcumin’s role in activating nuclear factor erythroid 2-related factor 2 (Nrf2) further enhances its antioxidant defense, boosting the expression of endogenous antioxidant enzymes such as superoxide dismutase (SOD) and glutathione peroxidase (GSH-px). This activation not only shields the mucosal cells from oxidative damage but also accelerates the healing process by reducing inflammation and promoting cellular repair mechanisms.

By inhibiting fibroblasts and myoblasts (collagen types I and III) of the oral mucosa, it reduces the onset of scar fibrosis. In combination with capsaicin, it induces cell apoptosis with increased CD8 T cells [73]. Because of the way it photosensitizes when oxygen is present, it can support photodynamic therapy in analgesic therapy [74]. Curcumin, by promoting the release of TGF-β-1, stimulates fibronectin and collagen production by fibroblasts, thereby facilitating re-epithelialization and mucosal healing. Curcumin activates Nrf2 and promotes the release of antioxidant enzymes such as SOD, catalase (CAT), glutathione (GSH), and GSH-px [45,75,76,77,78].

Curcumin has been observed to stimulate vascular endothelial growth factor (VEGF) and epidermal growth factor (EGF), both of which are responsible for cell proliferation and activity, thus promoting faster tissue healing [76,79,80,81].

### 1.2. Limitations of Curcumin

Despite its promising therapeutic potential, curcumin suffers from certain limitations, primarily related to its bioavailability. Curcumin is not hydrophilic and is poorly bioavailable. It is poorly absorbed from the gastrointestinal tract and is rapidly metabolized and eliminated in the gastro intestinal tract and liver [82,83,84]. To improve absorption and bioavailability, different formulations were used (emulsions, nanoparticles, hydrogels, ointments, tablets, chewing gum, powder) or it was used combined with other substances (piperine, honey, triamcinolone hyaluronidase gel, green tea, photodynamic therapy, tulsi). No side effects were observed even at high doses. A total of 12 g/day for 3 months was discovered to be a well-tolerated dosage [85,86,87,88,89,90]. The purpose of this review was to confirm the therapeutic properties of curcumin in RTMO and CTMO. However, further research is needed to better study its properties and develop proper protocols to better enhance its efficacy.

## 2. Materials and Methods

### 2.1. Search Strategy Literature

Our systematic review was conducted in accordance with the PRISMA (Preferred Reporting Items for Systematic Reviews and Meta-Analyses) guidelines. The literature search was performed using three major databases: PubMed, Web of Science, and Scopus. We searched for studies published within the last ten years, using the following key-words: “curcumin AND (oral mucositis OR oral disease) AND (radiotherapy OR chemotherapy).” The search focused on clinical trials and studies examining curcumin’s role in managing oral mucositis in cancer patients undergoing chemotherapy or radiotherapy. The final search was completed on 16 June 2024, and the review protocol was registered on PROSPERO with code ID “CDR 575864” [91].

### 2.2. Eligibility Criteria

The eligibility of the studies was determined by the following factors:

Population: oncology patients receiving chemotherapy and/or radiotherapy and experiencing oral mucositis.

Intervention: the use of curcumin or its derivatives for the prevention or treatment of oral mucositis.

Comparators: placebo, other standard treatments, or no treatment.

Outcomes: severity and duration of oral mucositis, pain relief, healing time, and adverse effects.

Study design: randomized controlled trials (RCTs), clinical trials, and pilot studies.

### 2.3. Inclusion Criteria

To be included in the review, studies had to meet the following criteria:

Full-text articles published in English.

Clinical trials with human participants, including randomized controlled trials, pilot studies, and comparative studies.

Studies specifically addressing the effects of curcumin on oral mucositis related to cancer treatments (chemotherapy or radiotherapy).

Studies providing quantitative outcomes related to mucositis severity, healing time, or pain management.

### 2.4. Exclusion Criteria

Studies were excluded if they met any of the following criteria:

In vitro or animal model studies.

Literature reviews, meta-analyses, and case reports.

Studies without clear quantitative data or that did not focus on the use of curcumin for oral mucositis in cancer patients.

Non-English articles or studies lacking a full-text version.

### 2.5. Selection of Studies

The selection process followed a step-by-step review of all identified articles. After an initial screening based on titles and abstracts, duplicate articles were removed. The remaining articles were assessed for relevance and eligibility through full-text reviews. Two authors (I.T. and L.F.) independently evaluated the studies, and any disagreements were resolved through discussion. The references of the included studies were also reviewed to identify additional relevant papers.

### 2.6. Statistical Analysis

A descriptive synthesis was used to analyze the included studies. Where applicable, data from the studies were summarized and compared regarding the effectiveness of curcumin on oral mucositis, its safety profile, and patient outcomes. Given the variability in the designs and interventions of the included studies, a meta-analysis was not performed. However, the findings were synthesized narratively, focusing on the key results and methodological differences [92].

## 3. Results

**Figure 5:**
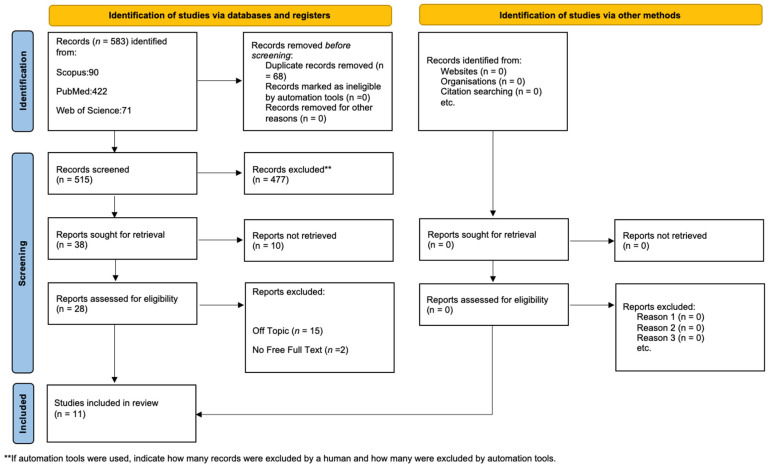
PRISMA flowchart.

Initially, the literature search turned up 583 documents: 422 from PubMed, 71 from WOS, and 90 from Scopus. After having removed 68 duplicates, 515 articles remained for assessment. Following the evaluation for eligibility and inclusion criteria, 17 papers were included in the final analysis. Figure 5 provides a summary of the study procedure and PRISMA flowchart, while Table 1 contains in-depth descriptions of the included papers.

### Quality Assessment and Risk of Bias of Included Articles

The quality of the included studies was assessed using the Cochrane Risk of Bias Tool (RoB 2) for randomized controlled trials (RCTs), which is designed to evaluate potential biases in five domains: randomization process, deviations from intended interventions, missing outcome data, measurement of the outcome, and selection of the reported results. Each study was rated as having either low risk, some concerns, or high risk of bias based on these criteria.

**Figure 6:**
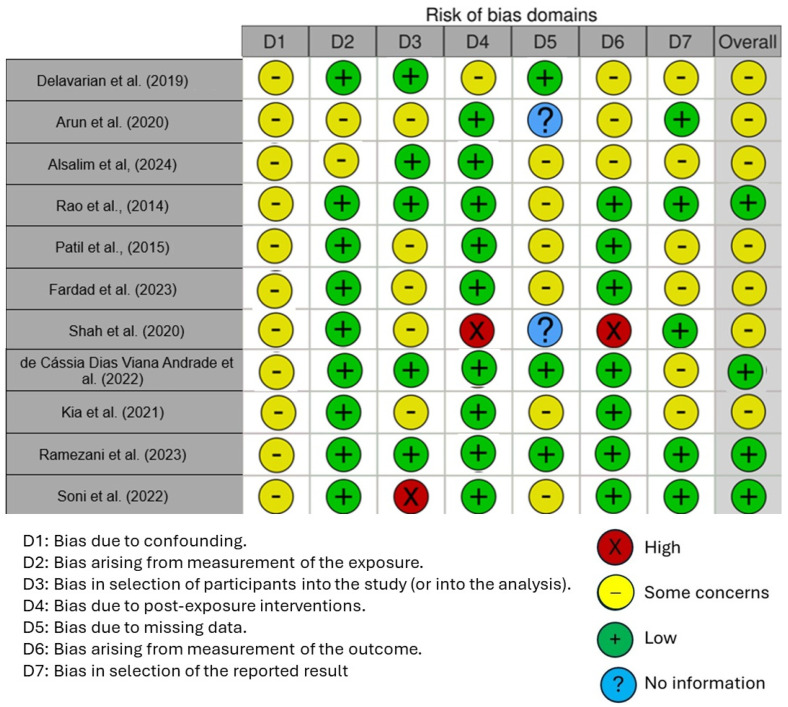
Risk of bias [7,58,62,63,65,91,93,94,95,96,97].

Figure 6 displays the risk of bias in the included studies. Most research raises some concerns about bias resulting from confounding. One parameter with a minimal risk of distortion is the measurement’s distortion. Because of participant selection bias, many studies have a low risk of bias. There is minimal risk associated with post-exposure distortion. The bias resulting from missing data is minimal in much research. Measuring outcomes has less bias. Most of the research also shows minimal selection bias in the published results. Five studies had a low risk of bias, according to the results, while seven raised some concerns.

## 4. Discussion

Anticancer therapy is widely recognized for its capacity to eliminate cancer cells, and it encompasses many treatments, such as radiotherapy and chemotherapy. An increased synthesis of free radicals, particularly reactive oxygen species (ROS) and reactive nitrogen species (RNS), is a notable adverse impact of these treatments [98]. Indeed, these unstable chemical compounds can harm both malignant and normal cells, since they are produced in high amounts during therapy, and can mediate lipid peroxidation, protein oxidation, and the production of double-strand breaks in DNA. These are some of the mechanisms that lead to toxic consequences in normal tissues, as well as to debilitating disorders, such as oral mucositis (OM) [99]. Since radiotherapy uses ionizing radiation, which interacts with H_2_O molecules in tissues to cause oxidative stress, an overabundance of ROS/RNS is frequently produced in response to anticancer treatment. On the other hand, certain chemotherapeutic medicaments directly increase the production of free radicals [100]. These processes underscore the need for measures to reduce the oxidative damage caused by cancer therapy, since they not only make treatment more difficult but can also result in therapy interruptions due to severe adverse effects.

Several research studies investigated how defense systems against oxidative stress, especially in the brain, are modulated by the nuclear factor erythroid 2 p45-related factor (Nrf2). Under normal circumstances, the leucine zipper protein (bZIP) Nrf2 is mostly found in the cytoplasm of the cells and, in order to start the transcription of protective genes in response to stress, Nrf2 translocates to the nucleus and attaches to DNA sequences, such as antioxidant response elements (ARE). Although Nrf2 activation is essential for oxidative stress defense (as already stated), overactivation of this protein is linked to a poor prognosis in a number of cancer types. It has been demonstrated that curcumin activates Nrf2, improving the effectiveness of cancer treatments by increasing heme oxygenase-1 (HO-1) and decreasing the Nrf2 inhibitor Keap1.

Additionally, curcumin’s modulatory effects on the Nrf2 signaling pathway improve insulin resistance due to its anti-inflammatory and antioxidant properties, while reducing metabolic reactions that cause inflammation and oxidative stress and promoting glutathione production by activating the Nrf2-HO-1 and Nrf2-Keap1 pathways. Other studies have confirmed the protective role of curcumin through Nrf2 regulation [101].

**Figure 7:**
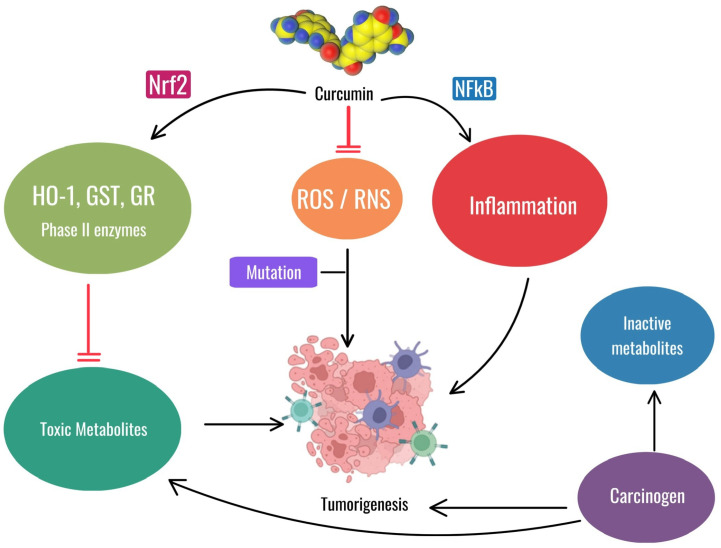
The image illustrates the dual role of curcumin. In the context of oral mucositis, curcumin reduces oxidative stress (ROS/RNS) and inflammation, aiding in the management of this non-cancerous condition. In a different context, curcumin can prevent tumorigenesis by activating Nrf2, reducing toxic metabolites and inhibiting NF-kB-mediated inflammation, thus counteracting mutations and tumor growth. Chlorhexidine mouthwash is one of the traditional treatments used today, but this agent causes problems by itself while being poorly effective. Newer treatments, such as hyaluronic acid-based formulations (mucosamin) and curcumin extracts of turmeric, are being explored for their potential benefits in treating this condition [17,102].

Shahcheraghi SH et al. also described these features with the observation that, similarly to curcumin, the Zinc-curcumin (Zn(II)-curc) compound has been shown to activate Nrf2, leading to anticancer effects through various mechanisms, such as increasing levels of the proteins HO-1, p62/SQSTM1, and Nrf2 itself, while reducing the levels of its inhibitor, Keap1. This interaction between p62/SQSTM1 and Nrf2 could be leveraged to improve cancer therapy outcomes [101]. The antioxidant and anti-inflammatory properties of curcumin may be useful for both oral mucositis and prevention of tumorigenesis, but their applications differ. For OM, curcumin’s role as an antioxidant and anti-inflammatory agent helps reduce the severity of oral mucositis by mitigating oxidative stress and inflammation caused by chemotherapy and radiation therapy. This relieves the painful symptoms of mucositis without interfering with the antitumor efficacy of these treatments. For the prevention of tumorigenesis, activation of Nrf2 and inhibition of NF-kB by curcumin are critical in reducing carcinogenesis by reducing inflammation and oxidative stress, thereby preventing mutations and tumor growth (Figure 7).

Inhibiting cancer development may involve reducing oxidative stress by activating antioxidant defenses, restoring tumor suppressor proteins, and modulating inflammation. Curcumin, over time, significantly influences Nrf2-mediated antioxidant enzymes and the tumor suppressor protein p53. On the other hand, inhibiting Nrf2 signaling decreases phase II antioxidant enzymes and p53 levels, while increasing inflammatory signals, such as TGF-β, and altering iNOS and COX2 regulation in lymphoma-bearing mice [101].

Curcumin shows great promise as a treatment therapy for OM and, since OM is a process involving several molecular and cellular events, the therapy needs to be multifactorial. Cancer treatments cause injury to the epithelial cells of the oral mucosa, leading to activation of factors such as TNF-α and IL-6, which are pro-inflammatory. Curcumin could modulate inflammation and tissue damage by inhibiting the transcription factor NF-κB, which regulates the synthesis of inflammatory cytokines. In addition to reducing inflammation, curcumin possesses powerful antioxidant properties that enable it to neutralize ROS, reducing oxidative damage to mucosal cells. This is particularly important, since oxidative stress amplifies inflammation, aggravating mucositis. In addition, curcumin stimulates tissue regeneration by increasing the levels of growth factors such as EGF, which are essential for the repair of damaged mucosa. This accelerates the healing of oral ulcers and reduces the overall duration of OM.

Another significant aspect is curcumin’s ability to modulate the apoptotic pathway, protecting epithelial cells from premature death and maintaining mucosal integrity, thus reducing the severity of ulcers. In addition, curcumin also shows potential as an antimicrobial agent, a relevant aspect in the management of mucositis, where secondary infections may further complicate the clinical picture. By reducing the microbial load in the oral cavity, curcumin helps prevent infectious complications, improving healing. Despite its benefits, curcumin has historically suffered from low bioavailability, limiting its clinical efficacy. However, recent innovations, such as nano-micelles formulations, have significantly improved its ability to reach target tissues, enhancing its therapeutic effects against mucositis and making it a more practical and effective option.

In a study by Delavarian et al., the use of a nanocurcumin oral dosage was evaluated in patients with radiation-induced OM for the treatment of head and neck cancer. RT induces OM as a very common and painful complication in basically all patients with head and neck cancers and is associated with other complications, including mucositis, which results in the inability to eat and an increased risk for infection [56,62,103,104]. A total of 32 patients were involved in the study, and a double-blind, randomized clinical trial was performed to assess the efficacy of SinaCurcumin^®^, formulated as a nanomicelles to enhance the bioavailability. The results showed that the occurrence of OM in the experimental group was delayed and the intensity was less compared to the control group that received the placebo. There were no significant side effects with nanocurcumin reported in this study’s administration information, and this suggests that it is safe for use and an effective medication for OM. No such reports of the clinical use of orally administered nanocurcumin in the radiation-induced type of OM were previously available. Therefore, this seems to be the first clinical report of the same, which documents the status of nanocurcumin in management and chewing/applicational prospects as a proved analogue for cancer therapy. Further studies with more numbers of cases and favorably designed dosing are warranted to prove this inference and optimize treatment protocols [62].

In the study by Arun et al., SCC of the head and neck is presented as one of the most common cancers in India, accounting for 12% of cases. The conventional methods to treat it include surgery, radiotherapy, and chemotherapy, each of which is often followed complications, including the major one, radiation-induced mucositis, which may cause therapy to be interrupted. Several agents may be used to treat it, but no single agent is approved by the FDA. This study aimed to assess whether turmeric extract, containing curcuminoids, would have the potential to decrease the severity of mucositis [93,105,106]. Curcumin has anti-inflammatory properties, but its oral bioavailability was found to be low. This trial utilized a bioenhanced form of curcumin, BCM-95, combined with essential oils for better absorption. In the randomized, single-blinded clinical trial conducted at R L Jalappa Hospital and Research Centre, Kolar, India, 64 patients with advanced head and neck SCC undergoing post-operative radiotherapy, chemoradiotherapy, or concurrent chemoradiotherapy were included. The subjects were randomized for a test group receiving 1.5 gm/day of turmeric extract capsules against the control group receiving placebo capsules. The results showed that from the third week of the treatment, the severity of mucositis was significantly lower in the turmeric group compared to the control group. Two months after follow-up post-treatment, the majority of the turmeric group had only mild mucositis compared to the control group, which were classified under a higher grade of mucositis [41,107,108]. It was concluded that with no systemic toxicity, the turmeric extract reduced the severity of mucositis and hence was quite effective as a mode of treatment in patients undergoing radiotherapy or chemoradiotherapy. The study emphasizes the possible therapeutic benefits of turmeric extract in the management of radiation-induced mucositis in neck and head cancer patients [93,109].

In 2024, Sarah Adnan Alsalim and others conducted a study to evaluate curcumin oral gel efficacy in relation to RIOM and its connection with salivary EGF. They divided thirty-one patients undergoing radiation in the therapy of neck and head tumors into one of two groups. One received curcumin, while the other was controlled by a magic solution. The second group consisted of a gel formulation which contained 10 mg of Curcuma longa extract, applied three times a day [110]. The difference noted here is that, when compared to the magic solution group, the patients in the curcumin group had a significant difference in WHO and VAS scores regarding the level of mucositis and pain. Moreover, at irradiation and at the end of irradiation, the level of salivary EGF was significantly elevated in the curcumin group. The conclusion of the study was that the curcumin gel appreciably reduced the severity of RIOM and related pain. This can be due to the high levels of EGF, which show a better healing response of the mucosa and control over the injury processes. Further studies involving a larger sample size should be conducted to replicate the findings [63].

In a study, Rao et al. have evaluated the effectiveness of turmeric in the mitigation of RIOM in patients suffering from head and neck malignancies. The common painful side effect of the radiotherapy, oral mucositis, is usually associated with a poor quality of life in ailing patients and reduced adherence to therapy [94,111].

In this study, 80 patients were randomly assigned to use a turmeric gargle or a povidone-iodine gargle during radiation treatment. The results indicated that the turmeric gargle significantly retarded the initiation of OM and reduced the level of its severity compared with the povidone-iodine gargle. Patients who used turmeric had fewer instances of severe mucositis, fewer breaks in treatment, and less weight loss. These results suggest that turmeric can be a very effective and inexpensive solution for the management of radiogen-induced mucositis [94,112,113,114].

In the pilot study conducted in 2015, Patil et al. evaluated the role of curcumin mouthrinse in the management of radio-chemotherapy-induced OM in patients with malignancy. This included 20 patients under treatment using radio-chemotherapy, who were divided randomly into two groups: one using curcumin mouthrinse and another using a standard chlorhexidine mouthwash. The patients’ symptoms of OM were rated at three different time periods: at the beginning, after 10 days, and after 20 days. The evaluation was performed on the WHO scale, OMAS, and NRS [58,115,116]. The results showed that the curcumin mouthrinse fared much better than chlorhexidine in reducing pain, erythema, and ulceration. The scores for pain NRS, erythema levels, and ulceration severity showed a significant improvement with the use of the curcumin mouthrinse, and the differences were statistically significant. Moreover, very minimal adverse effects and excellent compliance were proven in all patients treated with the curcumin mouthrinse in contrast to those who were treated with chlorhexidine. It is therefore clear that curcumin mouthrinse is not only more efficient but also safer in the treatment of OM associated with cancer therapy. The present study thus confirms that curcumin mouthrinse could be a very promising agent for the improvement of quality of life in patients undergoing radio-chemotherapy [58,117,118].

In a randomized, double-blind trial, Fardad et al. evaluated the efficacy of chlorhexidine mouthwash, mucosamin spray, and curcumin gel in treating chemotherapy-induced oral mucositis in patients with malignancy. Their results showed that all three products were effective, but the curcumin gel showed faster and more complete recovery, with fewer side effects. These findings suggest that curcumin might turn out to be an adjunct of value in oral mucositis; however, studies with larger sample sizes are recommended for the confirmation of results [7].

This is a dose-dependent painful syndrome in individuals receiving radiation treatment for cancers of the head and neck. With a view to preventing and managing RIOM, Shah et al. compared the efficacy of a mouthwash containing 0.1% curcumin using nanoparticles with another one containing 0.15% benzydamine. Curcumin showed promise in deferring the onset of RIOM by a fortnight, though both mouthwashes were equally efficacious in reducing the severity of the condition. Although this was a triple-blinded, randomized controlled study, the following were its limitations: small sample size and high loss to follow-up. Curcumin was found to be as safe and efficacious as benzydamine, with an added advantage of delaying the onset of RIOM. Curcumin mouthwash could be a safe and effective option compared to benzydamine in the management of RIOM, delaying its onset. Further studies, with larger sample sizes and at varying doses of curcumin are further indicated to strengthen the findings [95].

The article by Rita de Cássia Dias Viana Andrade et al. describes the comparative study of two kinds of treatment in the management of OM: photobiomodulation and aPDT mediated with curcumin. The results showed that PBM and aPDT decreased the severity of mucositis and the associated pain. Clinical improvement was observed earlier in the aPDT group compared to the other groups: the PBM and control groups. PBM was outstanding, as it improved the tissue healing process and reduced inflammation. On the other hand, aPDT was more effective at reducing the load of Candida yeast. This suggests that both modalities can improve the quality of life of patients with oral mucositis, since it is a non-invasive therapy with antimicrobial, analgesic, and anti-inflammatory effects [91].

According to Kia et al., the text describes how chemotherapy and radiotherapy used during cancer treatment often led to OM. Traditional treatments offer relief but do not prevent OM. Curcumin is a polyphenol with anti-inflammatory properties extracted from turmeric. However, the bioavailability of curcumin is poor, and thus its effectiveness has been improved by the preparation of curcumin nanomicelles. In a clinical trial, it was observed that patients receiving nanomicelle curcumin demonstrated less severe OM conditions and pain as compared to placebo counterparts, which indicated its effectiveness [96].

In a single-blind randomized clinical trial, Ramezani tested the efficacy of oral and topical formulations of curcumin in reducing the severity and pain of radiotherapy-induced ROM. Both formulations of curcumin significantly reduced the symptoms of ROM when compared with the placebo group, though no significant difference was seen between the two groups receiving the curcumin formulations. The anti-inflammatory property of curcumin was brought out as useful in the treatment of ROM and other radiation-related conditions. The study had its limitations, including the small sample size, in part due to the pandemic caused by COVID-19. Future research is needed for replication of the findings with higher levels of curcumin dosages and larger samples [65,119].

In this regard, Soni evaluated the role of a bio-enhanced formulation of turmeric on oral mucositis, which represents one of the common and serious side effects of chemoradiotherapy in head and neck cancer patients. The patients were selected from 60 individuals with cases of oral cancer after radical surgery. These patients were randomized into three groups and received daily either a low dose of 1 g/day or a high dose of 1.5 g/day of BTF or placebo during chemoradiotherapy treatment for six weeks [97,120,121,122]. The study was undertaken to evaluate the role of BTF on a variety of treatment-related toxicities, which includes, among others, dermatitis, oral mucositis, dysphagia or trouble swallowing, mouth discomfort, and weight loss. It was found that, compared with the placebo group, patients who received BTF had lower incidences of severe grade 3 oral mucositis, pain, dysphagia, and dermatitis. The incidence of grade 3 OM was reduced to 25% and 20% for the low and high dose BTF groups, respectively, from 65% in the placebo group. Likewise, grade 3 oral pain, dysphagia, and dermatitis were significantly reduced in the BTF groups. Furthermore, the patients who consumed BTF had lower weight loss compared to their placebo counterparts and also showed greater compliance with treatment. The study concluded that BTF (BCM-95) decreased the severity of oral mucositis, dysphagia, oral pain, and dermatitis associated with chemoradiotherapy in patients suffering from oral malignancy, thus opening up a potential avenue of support for cancer therapy [97,123,124,125,126].

## 5. Conclusions

Curcumin, a compound derived from turmeric, has shown significant promise in treating RIOM in patients with head and neck cancer, especially in its enhanced forms, such as nanocurcumin and BCM-95. These formulations significantly reduce the severity and pain of mucositis by improving bioavailability and amplifying curcumin’s antioxidant effects. Curcumin’s ability to neutralize ROS plays a key role, reducing oxidative stress, preventing further damage to the oral mucosa, and promoting healing. Studies have shown that curcumin can outperform conventional treatments, such as or chlorhexidine mouthwash, or placebo, improving the quality of life for patients and allowing them to continue cancer treatments without interruptions. Its tolerability is another advantage, as patients experience few side effects, even in its enhanced forms. However, despite these promising results, further research is needed to fully establish curcumin’s role in cancer therapy and RIOM management. This should focus on larger clinical trials, more diverse patient populations, and optimized dosing protocols to ensure consistent and reproducible outcomes. While innovative formulations, such as nanocurcumin, have significantly improved bioavailability, curcumin still faces limitations in terms of its therapeutic potential, particularly when administered in traditional forms. Future studies may explore personalized treatment approaches that incorporate curcumin alongside other complementary therapies, as well as the development of multi-targeted strategies for managing mucositis and other side effects of cancer therapy. The continued investigation into the molecular mechanisms behind curcumin’s anti-inflammatory and antioxidant actions could also uncover new therapeutic pathways, broadening its applications not only in RIOM but also in cancer prevention and adjunctive treatment. In conclusion, curcumin represents a highly promising natural compound with multiple potential applications in cancer therapy, particularly in the management of RIOM. Its continued development and the optimization of delivery methods hold the potential to improve patient outcomes and reduce treatment-related complications, making it an important focus for future research in oncology.

## Figures and Tables

**Table 1:** Examined articles.

Authors	Type of Study	Number of Patients	Aim	Materials and Methods	Conclusions
Delavarian et al. (2019) [62]	Double-blind, randomized clinical trial	32	To analyze how curcumin nanomicelle affects OM in patients with HNC undergoing radiation therapy.	Nanocurcumin (80 mg/day) vs. placebo during radiotherapy for head and neck cancer; assessed weekly for OM severity.	Nanocurcumin significantly reduced the severity of OM compared to placebo, with no notable side effects. It is an effective approach for preventing or mitigating OM in these patients.
Arun et al. (2020) [93]	Randomized controlled trial	61	To evaluate turmeric extract for reducing mucositis.	Group A: 500 mg turmeric thrice daily; Group B: placebo; assessed weekly using CTCAE and WHO.	Turmeric extract reduces incidence and severity of mucositis.
Alsalim et al. (2024) [63]	Clinical trial	31	To compare curcumin gel vs. magic solution for RIOM and salivary EGF.	16 patients used curcumin gel, 15 used magic solution. Saliva samples analyzed for EGF; mucositis assessed by WHO and VAS scales.	Curcumin gel reduced RIOM severity and pain, increased EGF levels. More research needed.
Rao et al. (2014) [94]	Randomized Controlled Trial	80	To evaluate the effectiveness of turmeric gargle in reducing radiation-induced OM.	Patients were randomly assigned to use either a turmeric gargle or a povidone-iodine gargle during radiation therapy. OM onset and severity were monitored.	Turmeric gargle significantly delayed the onset of OM, reduced severity, and led to fewer severe cases, interruptions, and weight loss compared to povidone-iodine.
Patil et al. (2015) [58]	Pilot study	20	To assess curcumin mouthrinse for OM from radio-chemotherapy.	20 cancer patients were divided into two groups: curcumin vs. chlorhexidine mouthrinse. Symptoms were assessed at days 0, 10, and 20.	Curcumin was more effective than chlorhexidine in relieving OM with fewer side effects.
Fardad et al. (2023) [7]	Randomized, double-blind study	71	To compare the efficacy of three treatments—curcumin, mucosamin, and chlorhexidine—in the treatment of chemotherapy-induced oral mucositis.	Group A: received Mucosamin^®^ oral spray, four puffs daily for two weeks. Group B: received curcumin gel 0.5%, four times daily for two weeks. Group C: received chlorhexidine mouth rinse 0.2%, diluted 1:1, for one minute four times daily for two weeks.	Curcumin could be considered a promising adjunct therapy for OM in cancer patients, but further studies with larger sample sizes and varied concentrations of curcumin are recommended to validate these findings.
Shah et al. (2020) [95]	Pilot randomized controlled clinical study	74 head and neck cancer	To compare the effectiveness and safety of 0.1% curcumin mouthwash and 0.15% benzydamine mouthwash in managing radiation-induced oral mucositis.	Two groups were formed; one received 0.1% curcumin mouthwash, and the other received 0.15% benzydamine mouthwash.	Both mouthwashes were equally effective in preventing severe RIOM (severity score ≥ 3) after dichotomization.
Rita de Cássia Dias Viana Andrade et al. (2022) [91]	Clinical randomized study	30	To evaluate the effectiveness of blue LED and curcumin-mediated antimicrobial photodynamic therapy (aPDT) as an adjuvant treatment for oral mucositis in cancer patients undergoing chemotherapy or radiation therapy.	Three groups of patients were assigned: the control group, which received nystatin treatment; the PBM group, which received low-level laser therapy; and the aPDT group, which received treatment with 450 nm blue LED and curcumin photosensitizer.	The degree of mucositis and pain scores decreased in both the PBM and aPDT groups; however, the aPDT group was notable for showing earlier clinical improvement compared to the PBM and control groups.
Kia et al. (2021) [96]	Clinical trial	50	To investigate the effects of nanomicelle curcumin on OM caused by chemotherapy and head and neck radiotherapy.	Study Group: received curcumin nanomicelle capsules, 80 mg, twice a day. Control group: received a placebo, twice a day. Duration: 7 weeks.	Nanomicelle curcumin capsules were effective in the prevention and treatment of OM induced by chemotherapy and head and neck radiotherapy.
Ramezani et al. (2023) [65]	Randomized, placebo-controlled trial	45	To assess the effectiveness of oral and topical curcumin formulations in reducing the severity and pain of radiation-induced oral mucositis (ROM) in patients undergoing head and neck radiotherapy.	Curcumin mouthwash (0.1% *w/v*). Sinacurcumin soft gel containing 40 mg curcuminoids as nano-micelles (SinaCurcumin^®^40). Placebo mouthwash.	
Tej Prakash Soni et al. (2022) [97]	Randomized, double-blinded, placebo-controlled trial.	60	To assess the impact of a bio-enhanced turmeric formulation (BTF) on OM and other chemoradiotherapy-induced toxicities in patients with oral cancer.	Patients were administered BTF capsules at either a low dose (1 g/day), high dose (1.5 g/day), or a placebo, daily for 6 weeks during concurrent chemoradiotherapy.	The incidence of severe (grade 3) oral mucositis, pain, dysphagia, and dermatitis was significantly lower in the BTF groups compared to the placebo group. Patients in the BTF groups also experienced less weight loss and had better treatment compliance.

## Data Availability

Not applicable.

## Abbreviations

aPDT	antimicrobial photodynamic therapy
ARE	antioxidant response elements
BTF	bio-enhanced turmeric formulation
bZIP	zipper protein
CT	chemotherapy
EGF	epidermal growth factor
FDA	food and drug administration
GSH	glutathione
GSH-px	glutathione peroxidase
HO-1	heme oxygenase-1
HNC	head neck cancer
LED	light emitting diodes
Nrf2	nuclear factor erythroid 2 p45-related factor
NRS	numerical pain rating scale
OM	oral mucositis
OMAS	Oral Mucositis Assessment Scale
PBM	photobiomodulation
RIOM	radiation-induced oral mucositis
ROS	reactive oxygen species
RT	radiation therapy
SCC	squamous cell carcinoma
SOD	superoxide dismutase
VAS	visual analogue scale
WHO	world health organization
Zn(II)-curc	zinc-curcumin
